# Reconstruct the proximal radius with iliac graft and elastic intramedullary nail fixation after tumor resection

**DOI:** 10.1186/s12957-016-0964-8

**Published:** 2016-08-08

**Authors:** Bin Zhu, Jielai Yang, Dongdong Cheng, Xiaofan Yin, Qingcheng Yang

**Affiliations:** 1 Department of Orthopedics, Shanghai Jiao Tong University Affiliated Sixth People’s Hospital, No. 600, Yishan Road, Shanghai, 200233 China; 2 Department of Orthopedics, Minhang Hospital, Fudan University, No.170 Xinsong Road, Shanghai, 201199 China

**Keywords:** Elastic intramedullary nail, Bone tumor, Iliac autograft, Reconstruction

## Abstract

**Background:**

This study aims to introduce a novel technique in treating benign bone tumors of the proximal radius by elastic intramedullary nail fixation and iliac graft after tumor resection.

**Method:**

In this retrospective case series, the treatment outcomes of 17 patients with benign bone tumor involving the proximal radius were reported from January 2010 to August 2014. All the patients received reconstruction surgery with iliac graft and elastic intramedullary nail fixation after tumor resection. Pain scoring was assessed using the 0 to 10 numerical rating scale. The quality of life scoring was assessed using the SF-30 scoring system. In addition, functional outcome was assessed with the Musculoskeletal Tumor Society score and the Disabilities of the Arm, Shoulder, and Hand score.

**Results:**

The mean follow-up was 16 months (range, 10–22). The average bone consolidate time was 19.2 weeks (range, 16–24 weeks). The pre- and postoperative pain scores were 5.47 ± 1.58 and 1.18 ± 0.39, respectively. The pain symptom was significantly ameliorated after the operation (*t* = 13.50, *p* < 0.01). The pre- and postoperative and the quality of life scores were 48.29 ± 6.58 and 77.47 ± 5.89, respectively; the quality of life score was dramatically improved (*t* = −20.11, *p* < 0.01). The mean Musculoskeletal Tumor Society score was 83.41 % (range, 63–93 %) and the mean Disabilities of the Arm, Shoulder, and Hand score was 14.1 (range, 5.8–38.3).

**Conclusion:**

Taken together, the application of iliac graft and elastic intramedullary nail fixation after excision of lesions might be associated to a significant reduction of the pain and improvement of QOL (quality of life) and limb function of patients with benign bone tumors of proximal radius.

## Background

Bone tumors are rare, comprising less than 1 % of all cancers [1]. Regarding localization of musculoskeletal tumors, the long bones of the low extremity held primacy over all other localizations, and the radius was only involved in 3.7 % (benign 3.4 %, malignant 0.03 %) [2]. Primary bone tumors of the proximal radius are even rarer. Giant cell tumors of the proximal radius, for instance, took up only 0.5 % of all giant cell tumors as reported in an article [3, 4].

The proximal radius is an important stabilizer for resisting valgus, rotatory, and axial forces of the elbow and with the radial head forms part of the proximal radioulnar joint and is important for forearm rotation. In addition, 60 % of the upper limb load is transferred through the radiocapitellar joint [5, 6]. Injury of the proximal radius can lead to impairment of the function of forearm.

Reconstructing the proximal radius and restoring stability are considered the general principles in treating bone disease of the proximal radius. Inappropriate treatment will cause functional limitation of the forearm, especially restriction of joint movement in flexion/extension and pronation/supination [7]. Complications of bone damage in this place include elbow stiffness, avascular necrosis, nonunion, and overgrowth of the head of the radius [8–10].

Plate fixation is a commonly used method and the results are generally favorable [11–13]. However, failure of fixation and nonunion have also been described [14, 15]. Hardware prominence and soft-tissue adhesions, resulting in limited forearm rotation, loss of fixation, and painful crepitus, are associated with plate fixation and are frequent reasons for revision surgery [16]. In addition, it is a technically challenging procedure.

In 1980, Metaizeau et al. [17] described and popularized a method of treating displaced radial neck fractures by introducing a K-wire into the medullary canal of the radius and pushing it proximally until its point reaches the inferior aspect of the epiphysis to elevate and rotate to achieve an anatomic reduction. We learn from this method and focus on the treating of bone tumors of the proximal radius. After the tumor was removed, an iliac graft was applied to fill up the bone defect and an elastic intramedullary nail fixation was introduced into the medullary canal of the radius and pushed proximally through the grafted iliac bone to get an effective fixation and an anatomic reduction.

We present a series of patents who underwent iliac bone graft and intramedullary nail fixation after tumor resection with the purpose to introduce this novel technique and exhibit the functional outcome and quality of life (QOL) of this surgical treatment.

## Patients and methods

### Patients

The patients were got from the Shanghai Sixth People’s Hospital from January 2010 to August 2014. All of them were initially assessed at a multidisciplinary sarcoma meeting, evaluating the history, radiographs, and the results of biopsies. The type of surgery was determined through discussion.

Written informed consent was obtained from the patients, and the study was performed in accordance with the Declaration of Helsinki and approved by the ethics committee of Shanghai Jiao Tong University Affiliated Sixth People’s Hospital.

### Inclusion and exclusion criteria

The inclusion criteria were benign bone tumors with no invasion of the soft tissue or involving the radial head, lesion less than 5 cm, with or without pathological fracture, and adherence to treatment and follow-up instructions. The exclusion criteria were malignant bone tumor involving the radial head or soft tissue, lesion more than 5 cm, or cognitive issue.

### Surgical technique

After tumor resection was performed under general anesthesia, bone graft is harvested from the iliac crest and shaped to fit the bone defection. The inferior radial metaphysis is exposed through a short lateral incision 1 to 2 cm above the epiphyseal plate. The soft tissues are separated carefully, avoiding injury to the cutaneous branch of the radial nerve. The cortex is perforated, and a K-wire between 1.2 and 2 mm in diameter, with the last 1.5 cm bent more sharply, is induced into the medullary canal of the radius. Guide the wire with a handle and push it upward through the iliac graft to the inferior aspect of the epiphysis. Turn the wire around its long axis to 180 so that its point faces inwards. This produces a medial shift of the radial head. The iliac graft and the radial head are both fixed firmly by this wire. The distal end of the nail is cut 5 mm from the bone, and the incisions are sutured. The mean length of boney defect after tumor resection was 3.5 cm (range, 2–5 cm). The residual soft tissue was loosely opposed to reduce the dead space, and a drain was inserted for 24 h in both the proximal and the donor sites. A long arm plaster cast is used to keep the elbow immobilized for about 3 weeks.

All the patients began functional training 3 weeks after surgery when the plaster cast was removed. The wire was not removed until the bone had consolidated. Postoperative radiographs were needed to assess the quality of operation. The diagnosis was confirmed by pathological examination postoperatively.

### Follow-up

The patients were reviewed clinically and radiologically by the same surgeon every 4 weeks after surgery. Pain scoring and QOL scoring were carried out preoperatively and postoperatively. In addition, range of motion (ROM), stability, and strength of the elbows were evaluated separately.

To evaluate the function of the forearm, we used the Musculoskeletal Tumor Society score (MSTS) [18] and the Disabilities of the Arm, Shoulder, and Hand score (DASH) [19] at each review. The MSTS score is a clinician-based measure of function, pain, and emotional acceptance by the patient as a whole and includes the evaluation of dexterity, positioning of the hand, and ability to lift. Each category is scored between 0 and 5, with 0 indicating a poor and 30 an excellent outcome. The results are presented as a percentage. It was reported as a validated measure for patients with sarcoma of the upper limb [20].

The DASH is a patient-based measure of function, specific to the upper limb. The score ranges from 0, equating to no disability, to 100 indicating complete disability. Beaton et al. have demonstrated its validity in the assessment of disorders of the upper limb [21].

### Statistical evaluation

The data were compiled and analyzed using SPSS version 21.0. Continuous data were expressed as mean values and standard deviation. Comparisons between different time points were done using paired Student’s *t* test. A significant result was taken as *p* < 0.05.

## Results

**Fig. 1:**
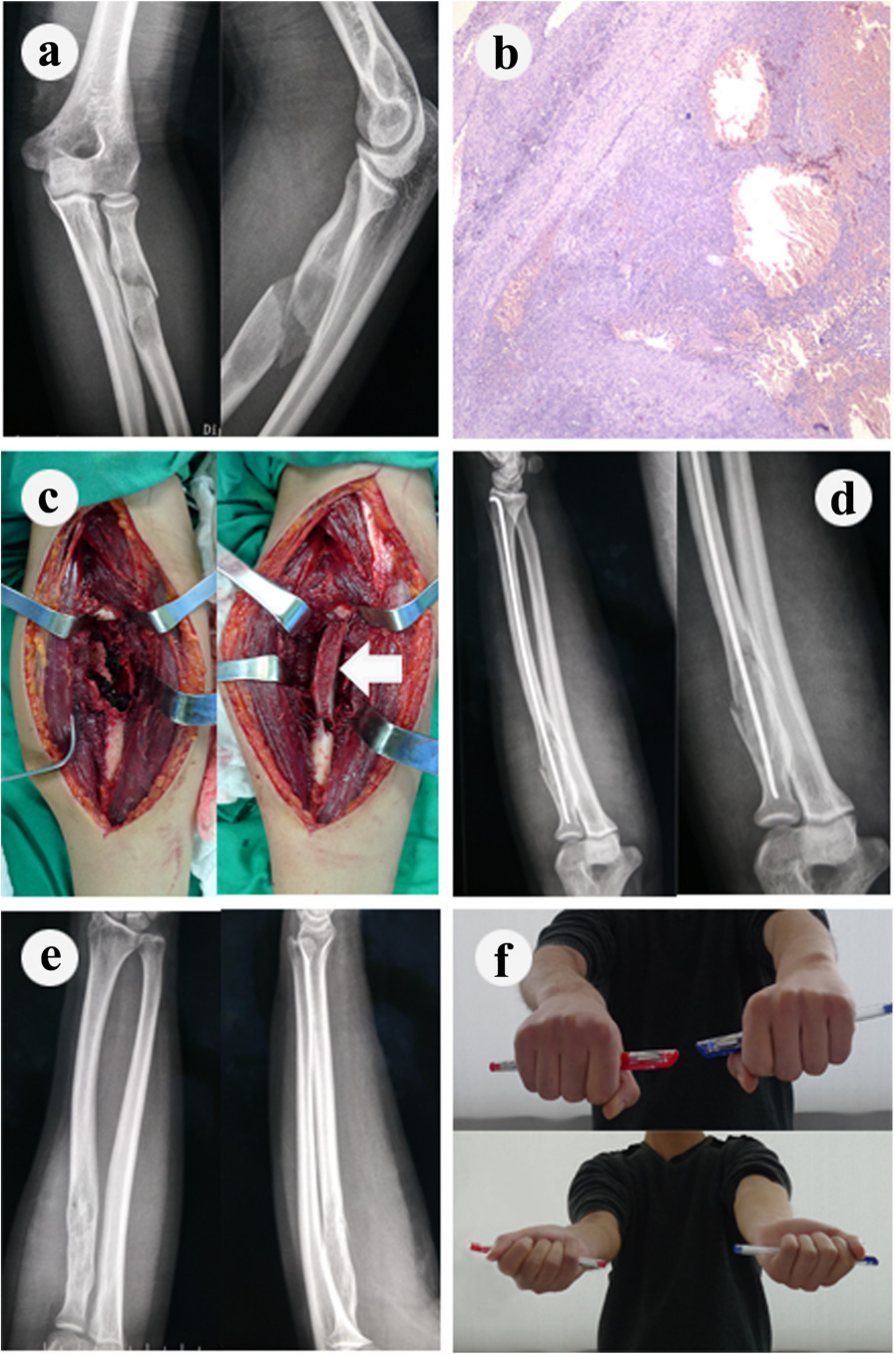
Case presentation (case 2). An 18-year-old man was diagnosed as having fibrous dysplasia (**b**) and pathological fracture (**a**) in the proximal radius of the dominant side. We reconstructed the proximal radius by iliac bone graft (*white arrow*) and elastic intramedullary nail fixation after tumor was removed (**c**, **d**). The nail was removed after he got boney consolidation 18 weeks after surgery. The radiological appearances (**e**) and the functional outcome (**f**) were satisfactory

Between January 2010 and August 2014, a total of 17 patients (9 male, 8 female) with benign bone tumors involving in the proximal radius were treated in our hospital with iliac graft and intramedullary nail fixation. Their mean age at the time of presentation was 30.6 years (range, 14–58 years). The complaints included simple pain in 5, pain and bump in 2, and pathological fracture in 10, with the dominant limb being affected in 9 patients (Table 1). The diagnoses confirmed by pathological examination are aneurysmal cyst (*n* = 6), fibrous dysplasia (*n* = 4), hemangioma (*n* = 1), eosinophilic granuloma (*n* = 1), simple bone cyst (*n* = 3), chondromyxoid fibroma (*n* = 1), and osteochondroma (*n* = 1). None of them experienced invasion of the radial head or the nearby tissue (Fig. 1).

**Table 1:** Details of the patients at latest follow-up

Case	Gender/age	Dominance side	Pathological diagnosis	Pathological fracture	Length of bone defect (cm)	Time to assessment (months)	Bone healing time (weeks)	Preoperative pain score	Postoperative pain score	MSTS (%)	DASH	Preoperative QOL score	Postoperative QOL score	Complications
1	M/24	Yes	ABC	Yes	2.5	16	16	5	1	87	10.8	54	88	None
2	M/18	Yes	FD	Yes	3	18	18	6	1	93	6.7	46	79	None
3	M/58	No	ABC	No	3	21	20	4	1	83	12.5	55	77	None
4	F/57	Yes	HD	Yes	2	10	20	4	1	80	14.2	48	71	radial nerve palsy
5	F/34	No	FD	No	5	13	16	7	2	80	11.67	40	69	None
6	F/16	Yes	EG	No	4	19	18	5	1	90	5.8	53	83	None
7	F/14	Yes	SBC	Yes	4	17	16	3	1	83	13.3	63	77	None
8	F/47	No	CF	Yes	3.5	14	22	9	2	63	38.3	38	72	None
9	M/22	No	OC	No	5	14	20	6	1	87	9.2	45	79	None
10	M/19	No	ABC	No	3	16	20	5	1	93	6.7	56	84	None
11	F/33	No	ABC	Yes	3.5	17	18	7	1	83	15	48	76	None
12	F/25	Yes	ABC	Yes	4	15	16	4	1	73	30.8	44	76	None
13	F/42	Yes	FD	No	4	15	22	5	1	90	7.5	43	83	None
14	M/15	Yes	SBC	Yes	3	22	20	5	1	93	5.8	50	78	None
15	M/30	No	ABC	Yes	2.5	13	18	6	1	80	15	47	73	None
16	M/24	Yes	FD	No	4.5	15	24	8	2	90	8.3	40	67	None
17	M/43	No	SBC	Yes	4	17	22	4	1	70	27.5	51	85	None

*F* female, *M* male, *ABC* aneurysmal bone cyst, *FD* fibrous dysplasia, *HD* hemangioma of bone, *EG* eosinophilic granuloma, *SBC* simple bone cyst, *CF* chondromyxoid fibroma, *OC* osteochondroma, *QOL* quality of life

### Surgical complications

All the patients were followed up for an average time of 16 months (range, 10–22 months). No patient was default in follow-up, and no local recurrence was found in any patients. Radial nerve palsy was found in one patient who recovered in 6 months after surgery. All the other patients were free of pain at the end of the follow-up, and no other obvious complications were noticed. No donor site morbidity was found.

### Result of evaluation

Bone healing was monitored each month through standard anteroposterior (AP) and lateral view radiograph. Primary union was achieved in all patients. The average bone healing time of the patients fixed by the elastic intramedullary nail was 19.2 weeks (range, 16–24 weeks).

The mean preoperative pain score was 5.47 ± 1.58, whereas the postoperative pain score was 1.18 ± 0.39. The symptom was significantly ameliorated after the operation (*t* = 13.50, *p* < 0.01). The mean preoperative QOL score was 48.29 ± 6.58, whereas the mean postoperative score was 77.47 ± 5.89. Therefore, the QOL dramatically improved (t = −20.11, *p* < 0.01) (Table 2).

**Table 2:** Pre- and postoperative pain and quality of life scores

	Preoperative	Postoperative	*t*	*p*
Pain score	5.47 ± 1.58	1.18 ± 0.39	13.50	<0.01
Quality of life	48.29 ± 6.58	77.47 ± 5.89	−20.11	<0.01

The result of ROM, stability, and strength are summarized in Table 3. There was no instability in external rotation or with varus and valgus stress of the affected elbow at the final clinical follow-up. The mean ROM was 116° (range, 90°–140°) of flexion/extension and 137° (range, 105°–160°) of pronation/supination.

**Table 3:** Result of the clinical evaluations performed at last follow-up

Case	Range of motion (°)		Stable	Power	
	Extension/flexion	Pronation/supination		Extension/flexion	Pronation/supination
1	0/130	80/80	Yes	5/5	5/5
2	5/130	70/70	Yes	4/5	4/5
3	5/120	70/80	Yes	5/5	5/5
4	0/120	70/70	Yes	5/5	4/5
5	20/110	55/60	Yes	4/4	4/4
6	10/130	60/60	Yes	5/5	5/5
7	5/130	90/80	Yes	5/5	5/5
8	20/120	60/65	Yes	5/5	4/4
9	0/140	70/60	Yes	5/5	5/5
10	5/130	80/65	Yes	5/5	5/5
11	5/120	65/70	Yes	5/5	5/5
12	10/130	55/60	Yes	5/5	5/5
13	15/130	70/70	Yes	4/5	4/5
14	10/120	70/60	Yes	5/5	5/5
15	10/130	70/70	Yes	5/5	5/5
16	15/110	50/55	Yes	4/4	4/4
17	5/120	90/80	Yes	5/5	5/5

At the end of follow-up, the mean MSTS score of the individuals fixed with intramedullary nail was 83.41 % (range, 63–93 %), with the lowest scores being for the emotional acceptance and lift. The outcome scores were good for pain and positioning of the hand (Table 4). The mean DASH score was 14.1 (range, 5.8–38.3). Patients got favorable overall outcome.

**Table 4:** The musculoskeletal tumor society scores at latest follow-up

Case	Time to assessment (months)	Pain	Function	Emotional acceptance	Hand positioning	Dexterity	Lifting ability
1	16	5	4	4	5	4	4
2	18	5	5	4	5	5	4
3	21	5	4	3	5	4	4
4	10	4	4	3	5	5	3
5	19	5	3	4	4	5	3
6	13	5	4	4	5	4	5
7	14	5	4	3	5	4	4
8	17	4	2	3	4	2	3
9	14	5	4	3	5	5	4
10	16	5	5	4	5	5	4
11	17	4	4	3	5	4	5
12	15	5	4	3	4	3	3
13	15	5	4	5	5	4	4
14	22	5	5	4	5	5	4
15	13	5	4	3	4	4	4
16	15	5	4	4	5	5	4
17	17	5	3	2	5	2	4

## Discussion

An average follow-up of 16 months (range, 10–22 months) allowed time for complications and outcomes to be assessed. The results suggested satisfaction with pain relief and QOL improvement. The mean DASH score of 14.1 represents moderate disability compared with the general population. Hunsaker et al. [22] established a mean score of 10.10 for the general population. And the mean MSTS score of 83.41 % indicates a favorable functional outcome, especially in positioning of hand and dexterity.

Injury of the proximal radius will damage the function of the forearm, including the range of the joint movement in flexion/extension and pronation/supination. Remodeling capabilities of the forearm are often unpredictable [23]. Many have advocated the simple rule that the younger the patient and the more distal the lesion, the better the result will be [24, 25]. In midshaft and more proximal lesion, more anatomic reduction is generally preferable, as remodeling may not occur, and residual deformity is more likely to affect function [26, 27]. Tarr et al. [28] reported similar findings of lost forearm rotation due to angular deformity. Reconstruction of the radius bone is quite necessary to keep from negative functional outcome of the forearm.

Multiple techniques have been used to reconstruct the proximal radius and improve functional outcome, including free vascularized fibular bone graft [29], bipolar-type floating radial head prosthesis [30], and vascularized iliac crest graft [31], while the outcome of these techniques have been variable. The surgery we carry out in treating bone tumors of the proximal radius by tumor resection, iliac bone graft, and elastic intramedullary nail fixation stems from the method of treating radial head fracture, which was described and popularized by Metaizeau et al. [17] in 1980. Wang et al. once reported 23 patients with radial neck fracture treated with this technique, and 15 patients got excellent and 6 patients got good outcome according to MEPI [32]. We learn from this method and make some improvements. Iliac bone graft is used to fill the bone defect in the proximal radius. We reported 17 patients in this paper with bone tumors involving in the proximal radius. All were treated with iliac autograft and intramedullary nail fixation after tumor resection. The result suggested satisfaction with pain relief, QOL improvement, and functional outcome.

Internal fixation with steel plants is a classical and most commonly used way in treating bone fractures. But it requires a bigger incision and may cause a severe injury of the soft tissue, which may destroy the periosteal blood supply and lead to avascular necrosis because the proximal epiphysis of the radius has no sufficient soft-tissue coverage [33, 34]. The function of the forearm may also be damaged and the risk of infection will be higher. Anderson et al. [35] once reported 330 fractures in 244 patients. Of these patients, 112 had fractures of both the radius and ulna, 50 had single fractures of the ulna, and 82 had single fractures of the radius. All the fractures were treated with compression plates. Of these, 321 (97.3 %) united (4 had delayed union), and 9 failed to unite. Seven (2.9 %) of the 244 patients had serious infections after surgery. Most importantly, the removal of the steel plate is also a major operation and brings the risk of infection as well. In our study, all the patients achieved bone healing at the end of follow-up. No infection or avascular necrosis was found. Our results demonstrated a more favorable overall outcome than steel plate fixation.

Radial nerve dysfunction is a common sequela of a diaphyseal humeral fracture and proximal radius fracture, or the surgery to repair them [36, 37]. Surgery places the radial nerve at risk usually because of traction, but sometimes due to pressure from a retractor, the exposure, or damage from a drill or implant [38]. Observational studies identified that plate fixation had a higher percentage of radial nerve palsy compared with intramedullary nail fixation [39]. In this study, only one patient got radial nerve palsy and recovered in 6 months. Surgical exposure might be the reason associated with this iatrogenic transient dysfunction. Attention should be paid to avoid much irritation to the nerve.

The autologous bone is used to help promote bone healing in fractures and to provide structural support for reconstructive surgery [40]. The results of autologous bone graft are more predictable than the use of xenografts, cadaveric allografts, or synthetic bone substitutes because autologous bone grafts provide osteoinductive and osteoconductive properties [41] and are not immunogenic. Autologous bone grafts harvested from the iliac crest are commonly used in reconstructive orthopedic surgery with an advantage that it is easy to obtain [42]. Cancellous bone becomes revascularized more easily and, therefore, heals more quickly than cortical bone [41]. However, donor site morbidity is always well documented. The rate of major complications requiring secondary intervention is reported to range from 2.5 to 39 % [43]. Major complications include neurological injury, vascular injury, deep infection, large hematoma, bowel herniation, fracture, or pelvic instability with impaired gait. The rate of minor complication, which resolves without any intervention, ranges from 10 to 40 % [43]. Minor complications reported include superficial infection, seroma, unacceptable cosmesis, and temporary paresthesia. In our study, all the patients got boney consolidate at the end of follow-up. The mean length of boney defect after tumor resection was 3.5 cm (range, 2–5 cm). No donor site morbidity was found in our series.

The proximal radius is a rare site for primary bone tumors. Papers describing management are therefore limited and include few patients. The results have been inconclusive, and different outcome measures have been used. No conclusions about the optimum form of treatment can therefore be drawn. This series is, however, relatively large and the overall outcome is relatively good. The results we got are more conclusive. With iliac autograft and intramedullary nail fixation after tumor resection, we not only reconstruct the proximal radius but also accomplished internal fixation within a smaller incision, which allows less damage on adjacent soft tissue and decreases the risk of radial head avascular necrosis.

Certain limitations of this study should be noted. It was retrospective and based on a small number of patients. In addition, the indications for this procedure are limited to those patients with no invasion of the radial head, making it difficult to accumulate large numbers. The time of follow-up was relatively short, and the functional outcome needs to be further investigated in studies with long-term follow-up.

## Conclusion

To sum up, the application of iliac autograft and elastic intramedullary nail fixation after excision of lesions might be associated to a significant reduction of pain and improvement of QOL and limb function of patients with benign bone tumors of proximal radius.
